# Evaluation of the Effects of *Schisandra chinensis* on the Myocardium of Rats with Hyperthyroid Heart Disease by Using Velocity Vector Imaging Combined with the Estimation of p53 Expression and Calmodulin Activity

**DOI:** 10.1155/2020/5263834

**Published:** 2020-07-30

**Authors:** Rui Hou, Xuanshun Jin, Yihua Gao, Dandan Sun, Weiping Ma, Puyin Sun, Chengzi Jin

**Affiliations:** ^1^Department of Ultrasonic Diagnosis, General Hospital, Tianjin Medical University, Tianjin, Tianjin 300000, China; ^2^Department of Cardiology, Yanbian University Hospital, Yanji, Jilin 133000, China; ^3^Department of Ultrasonic Diagnosis, Yanbian University Hospital, 1327 Juzi Street, Yanji, Jilin 133000, China; ^4^Department of Ultrasonic Diagnosis, Hainan General Hospital, Haikou, Hainan 570100, China; ^5^Key Laboratory of Marine Drugs, Chinese Ministry of Education, School of Medicine and Pharmacy, Ocean University of China, Qingdao, Shandong 266003, China; ^6^Department of General Surgery, Tianjin Hospital, Tianjin, Tianjin 300000, China

## Abstract

*Schisandra chinensis* (SC) is reported to improve myocardial ischemia. Velocity vector imaging (VVI) is a noninvasive technique for evaluating myocardial function in humans, while few reported on the application in animals. In this study, we aimed to evaluate the improved effects of SC on the myocardium of Sprague Dawley rats having hyperthyroid heart disease (HHD) using VVI technique. HHD models were established by injecting daily with subcutaneous levothyroxine (0.5 mg/kg). Then, the SC group was administered the aqueous extract of SC (2 g/kg) once daily, while the HHD and control (CON) groups were administered the same amount of distilled water daily. All the rats were provided the same amount of food and water daily, and the intervention was stopped after 28 days. The efficacy of SC in HHD rats was evaluated by ultrasound VVI. The serum total triiodothyronine level, total thyroxine level, N-terminal pro-brain natriuretic peptide expression, p53 expression, and calmodulin (CaM) activity were assessed by western blotting, Hematoxylin-Eosin and Masson staining, and electron microscopy. The results indicated that SC significantly improved the systolic velocity, diastolic velocity, strain, systolic strain rate, and diastolic strain rate of the heart by significantly reducing p53 expression and CaM activity (*P* < 0.05), improving myocardial fibrosis in HHD rats. Also, VVI can be a valuable tool for the evaluation of myocardial function in HHD rats.

## 1. Introduction

Hyperthyroid heart disease (HHD) resulting from a long-term elevated level of serum thyroxine is a common complication of hyperthyroidism, causing cardiac complications such as arrhythmia, cardiac hypertrophy, or myocardial fibrosis [1, 2]. Velocity vector imaging (VVI) is a noninvasive technique that uses ultrasound pixel speckle tracking and border tracking and calculates the trajectory of the myocardium as a vector for a comprehensive evaluation of the myocardial motor function [3, 4]. Compared to two-dimensional images, VVI adds information on the speed of the movement of tissues. Furthermore, compared to tissue Doppler, VVI reduces the angle of dependence and, therefore, is used as a valuable tool for the evaluation of myocardial function in humans, while few reported on the application in animals. Due to the availability of model animals and the difficulty of obtaining human specimens, clinical VVI technology is more difficult to detect on model animals and more difficult to be consistent with the basic research mechanism, which severely limits the application of VVI technology in basic medicine.

*Schisandra chinensis* (Turcz.) Baill (S. chinensis) is commonly known as “Beiwuweizi” in Chinese, and it has been used as a Chinese traditional herbal medicine for the treatment of many diseases. Its chemical components are mainly volatile oil, lignan, organic acids, polysaccharides, fatty oils, amino acids, pigments, and tannins. Previous studies found that *Schisandra chinensis* (SC) could improve myocardial ischemia and exert cardioprotective effect [5, 6]. Here, we aimed to determine the changes in myocardial function in HHD rats treated with SC using VVI technique, combined with the estimation of the expression of N-terminal pro-brain natriuretic peptide (NT-proBNP) and p53 protein and calmodulin (CaM) activity, so that VVI could be developed as a valuable tool for evaluating myocardial function in HHD animals.

## 2. Materials and Methods

### 2.1. Preparation of an Aqueous Extract of SC

SC was purchased from the Yanji New Drug Pharmacy (place of origin: Liaoning, China) and diluted in distilled water (1 : 10). After soaking for 20 min, SC water was heated, refluxed, and extracted for 45 min, twice. The extracts were freeze-dried after concentration under reduced pressure.

### 2.2. Experimental Animals and Model Preparation

Male Sprague Dawley rats (*n* = 30; 5-6 weeks; 180–200 g) were provided by the Laboratory Animal Center of Yanbian University. The animal experiments were approved by the Animal Experimental Ethics Committee of Yanbian University. The rats were allowed to acclimatize for one week and, then, randomly divided into a control group (CON; *n* = 10), HHD group (*n* = 10), and SC group (*n* = 10). The HHD and SC groups were injected daily with subcutaneous levothyroxine (0.5 mg/kg). The CON group was injected daily with the same amount of normal saline subcutaneously. The SC group was administered the aqueous extract of SC (2 g/kg) once daily, while the HHD and CON groups were administered the same amount of distilled water daily. All the rats were provided the same amount of food and water daily, and the intervention was stopped after 28 days.

### 2.3. Echocardiography Using VVI Technique

After interventions, transthoracic echocardiography was performed in all the groups of rats using the Siemens Acuson S2000 Color Doppler Ultrasound (Siemens Medical Solutions, Inc., USA) with a 10V4 probe (10 MHz). Data were saved in apical four-chamber heart sections. VVI analysis was performed offline, and the systolic velocity (Vs), diastolic velocity (Vd), strain (S), systolic strain rate (sSR), and diastolic strain rate (dSR) were obtained. VVI analysis of each rat was performed five times by a senior physician, and the mean of five experimental readings was recorded.

### 2.4. Serum Biochemical Estimation by Enzyme-Linked Immuno Sorbent Assay (ELISA)

Blood samples were collected from the abdominal artery of the rats. The total triiodothyronine (TT3), total thyroxine (TT4), and NT-proBNP levels in rat serum were estimated by using ELISA kits (TT3, TT4, and NT-proBNP ELISA kit no. GC–0607, GC–0608, and ml003242, respectively; Shanghai, China) according to the manufacturer's instructions. In brief, 100 *μ*L serum was collected and reacted with 50 *μ*L biotinylated antibody in the antibody-coated plates for 90 min at 37°C. Then, the microplates were washed and incubated with 100 *μ*L streptavidin-horse radish peroxidase (HRP) for 30 min at 37°C. 3,3′,5,5′-Tetramethylbenzidine (TMB) was added and reacted for 20 min. The absorbance of the solution was measured with a microplate reader (Pennsylvania, USA) at 450 nm.

### 2.5. Western Blot Analysis

Proteins were extracted from frozen rat heart tissues, and the protein concentration was determined by the bicinchoninic acid (BCA) method using radioimmunoprecipitation assay lysis buffer with henylmethanesulfonyl fluoride (PMSF) (Beijing Solarbio Science and Technique Co., Ltd., Beijing, China, no. P0100). Twenty micrograms of protein were subjected to sodium dodecylsulphate polyacrylamide gel electrophoresis (SDS–PAGE) (12% acrylamide) (Beijing Solarbio Science and Technique Co., Ltd.; no. P1200–50T; Beijing, China) and transferred on to a polyvinylidene membrane. After the membrane was washed with phosphate buffered solution containing tween-20 (PBST, pH 7.5) and blocked with 5% skim milk powder solution prepared with PBST solution, it was incubated for 12 h at 4°C with the primary antibody (dilution 1 : 2,000, Santa Cruz Biotechnique, Inc., Santa Cruz, CA, USA.). After the membrane was washed with tris buffered saline tween (TBST), it was incubated with the secondary antibody (dilution 1 : 10,000, BIOSS, Beijing, China) at 37°C for 1 h. A chemiluminescence detection system was used to evaluate p53 expression and CaM activity. The target protein levels were normalized by GAPDH (Quantity OneVersion 4.6.2).

### 2.6. Histopathological Examination

The rats were anesthetized by injecting 20% urethane solution (0.5 mL/100 g) intraperitoneally. The hearts were excised and weighed. The left ventricular myocardial tissues were fixed in formaldehyde. The paraffin sections were stained with hematoxylin-eosin (HE) and Masson's trichrome staining kit (Nanjing SenBeiJia Biological Technique Co., Ltd., Nanjing, China). Four randomly selected microscopic fields from each Masson's-stained section were analyzed for collagen deposition using Image-Pro Plus 6.0 software (Media Cybernetics, Inc., Bethesda, MD). The results were expressed as the collagen volume fraction (CVF), which was calculated as collagen area/total area × 100%. The myocardial tissues of the left ventricular apex (1 mm³) were fixed in glutaraldehyde, processed, and observed under an electron microscope (SEM, Model S-4200, Hitachi Ltd., Tokyo, Japan).

### 2.7. Statistical Analyses

All data are presented as mean ± standard deviation (SD) from the least three independent experiments. All data were analyzed using SPSS version 19.0 (IBM SPSS, Armonk, NY). All the data, except for the histomorphometric values, were statistically analyzed with one-way analysis of variance (ANOVA) followed by Tukey's post hoc test. The homogeneity of variance was checked using the Levene test. The histomorphometric data were statistically analyzed by either ANOVA + Tukey's post hoc test or the Kruskal–Wallis H-test + the Mann–Whitney *U* test (MW) (the nonparametric tests) depending on the results of the Levene test. Differences were considered statistically significant when the *P* value <0.05.

## 3. Results

### 3.1. Effects of SC on Myocardial Function as Evaluated by VVI

**Figure 1:**
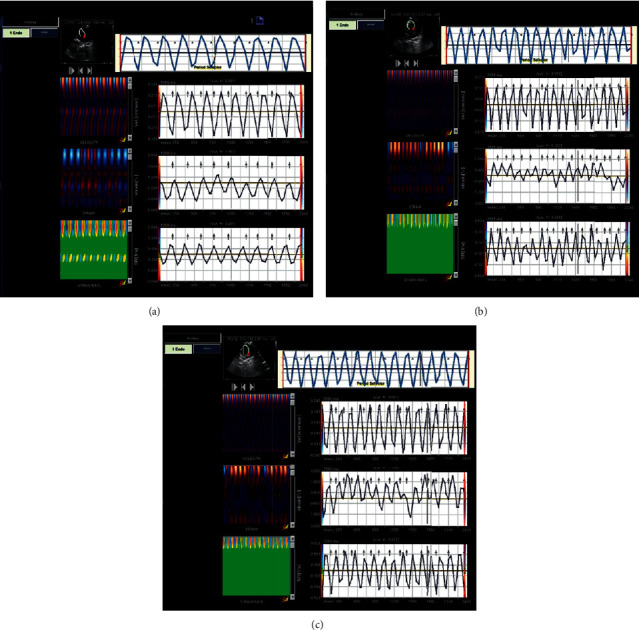
Echocardiographic velocity vector images of the experimental groups (CON, control group; HHD, hyperthyroidism heart disease model group; SC, *Schisandra chinensis* group; and VVI, velocity vector imaging) (5.16^*∗*^6.19 cm).

The myocardial functions of the three groups of rats were evaluated by VVI, and the results are summarized in Table 1. Compared to the CON group, the HHD group showed decreased systolic velocity (Vs), diastolic velocity (Vd), strain (S), systolic strain rate (sSR), and diastolic strain rate (dSR) (*P* < 0.05). Vs and S significantly differed among the groups (*P* < 0.01). The Vs, Vd, S, sSR, and dSR in the SC group were higher than those in the HHD group (*P* < 0.05). Vs and Vd significantly differed among the groups (*P* < 0.01) (Figure 1).

### 3.2. Effects of SC on Serum TT3, TT4, and NT-proBNP Levels of HHD Rats

**Figure 2:**
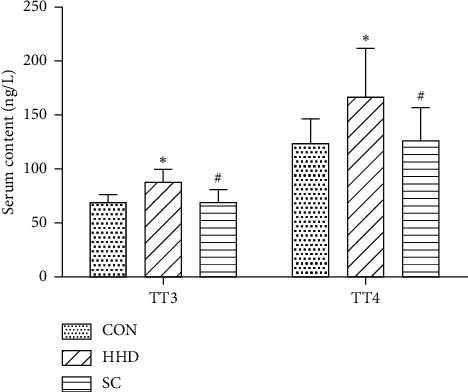
Serum level of total triiodothyronine (TT3) and total thyroxine (TT4). The results are expressed as the mean ± SD. ^*∗*^*P* < 0.05 vs. CON group; ^#^*P* < 0.05 vs. HHD group (CON, control group; HHD, hyperthyroidism heart disease model group; and SC, *Schisandra chinensis* group) (7.89^*∗*^13.89 cm).

**Figure 3:**
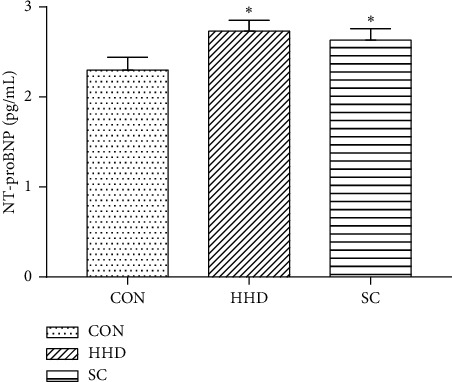
Serum level of N-terminal pro-brain natriuretic peptide (NT-proBNP). The results are expressed as the mean ± SD. ^*∗*^*P* < 0.05 vs. CON group (CON, control group; HHD, hyperthyroidism heart disease model group; and SC, *Schisandra chinensis* group) (6.58^*∗*^10.28 cm).

TT3 and TT4 are important serum markers of hyperthyroidism. Compared to the HHD group, the CON group showed increased levels of TT3 and TT4 (*P* < 0.05) in serum, whereas the SC group showed decreased levels of TT3 and TT4 (*P* < 0.05) in serum (Figure 2). The HHD and SC groups exhibited significant differences in the levels of NT-proBNP when compared to the CON group (*P* < 0.05) (Figure 3).

### 3.3. Effects of SC on p53 Expression and CaM Activity

**Figure 4:**
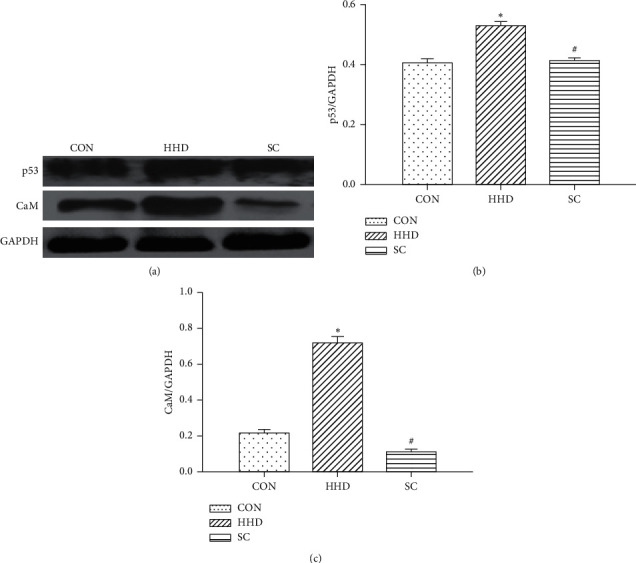
Expression of p53 and CaM in the three groups: control group (CON), hyperthyroidism heart disease model group (HHD), and *Schisandra chinensis* group (SC). (a) Western blot analysis of myocardial tissue. (b-c) Ratios of p53 and CaM to GAPDH. The protein levels were estimated by densitometry. The results are expressed as the mean ± SD. ^*∗*^*P* < 0.05 vs. CON group; ^#^*P* < 0.05 vs. HHD group (4.31^*∗*^8.56, 7.93^*∗*^12.8, 7.06^*∗*^10.02 cm).

The expression of p53 and CaM was determined by western blot analysis. Compared with the HHD group, SC significantly reduced p53 expression and CaM activity induced by levothyroxine (*P* < 0.05) (Figure 4).

### 3.4. Effects of SC on the Myocardial Structure by Histopathological Examination

**Figure 5:**
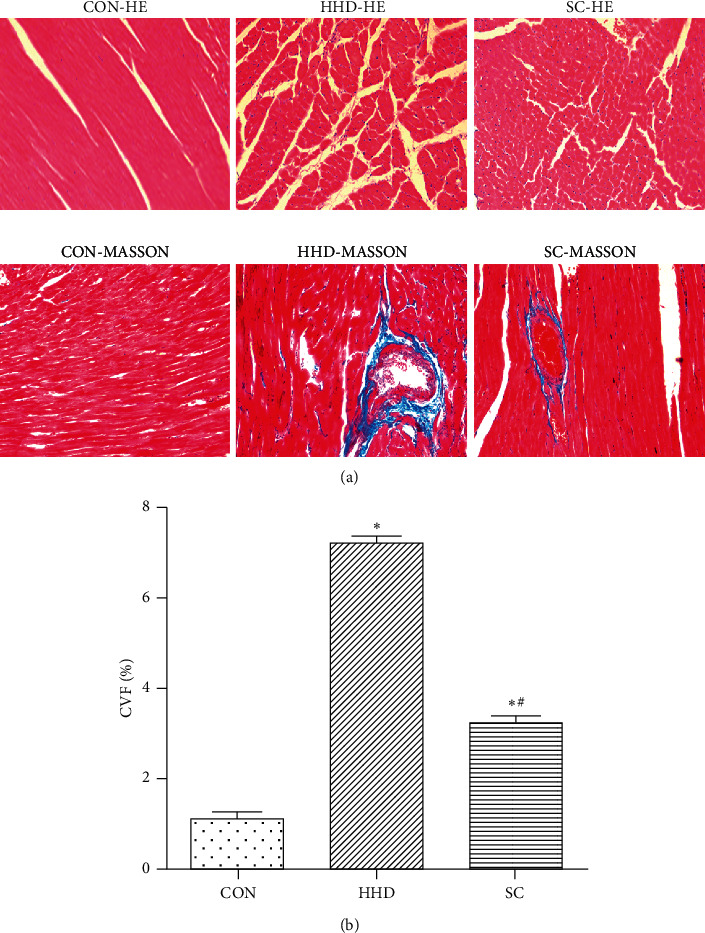
Hematoxylin and eosin and Masson's trichrome staining for the determination of the collagen volume fraction (CVF) of myocardial tissue (magnification, 200x). The results are expressed as the mean ± SD. ^*∗*^*P* < 0.05 vs. CON group; ^#^*P* < 0.05 vs. HHD group (CON, control group; HHD, hyperthyroidism heart disease model group; and SC, *Schisandra chinensis* group) (17.22^*∗*^14.64 cm).

HE and Masson's staining images of the myocardial tissues are shown in Figure 5. The morphology was normal in the CON group. The HHD group of rats displayed cardiomyocyte hypertrophy and thickening of the vessel wall with the disappearance of normal muscle fibers surrounding the vessel wall and replacement with fibrous scar tissue. A part of the heart muscle exhibited focal necrosis and fibrosis. The findings in the SC group revealed significant improvement in morphology, and the myocardial structure resembled that of the CON group. The myocardial CVF of the HHD group increased (*P* < 0.05) compared to that of the CON group, and the CVF of the SC group decreased (*P* < 0.05) compared to that of the HHD group.

### 3.5. Electron Microscopic Analysis of Rat Cardiomyocytes in the Three Groups

**Figure 6:**
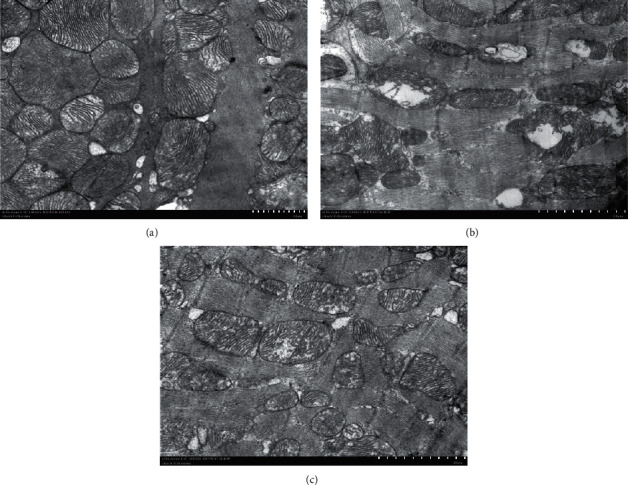
Structure of the myocardial fibers examined by electron transmission microscopy (magnification, 200x) (CON, control group; HHD, hyperthyroidism heart disease model group; and SC, *Schisandra chinensis* group) (20^*∗*^7.99 cm). (a) CON, (b) HHD, and (c) SC.

The structure of the myocardial fibers in the CON group was normal with dense mitochondria. The HHD group displayed disorders in the structure of the myocardial fibers with ruptured muscle fibers along with swollen and ruptured mitochondria. Compared to the HHD group, the SC group showed improvements in the structure of the myocardial fibers along with a reduction in the number of ruptured muscle fibers and swollen and/or ruptured mitochondria (Figure 6).

## 4. Discussion

In the present study, we investigated the feasibility of evaluating the myocardial movement in rats by using VVI. In addition, we determined the effects of SC on cardiac functions in a rat model of HHD induced by a long-term elevated serum thyroxine level. We found that the myocardial function in rats with HHD can be evaluated using VVI. Furthermore, SC was found to improve the myocardial dysfunction caused by HHD. The underlying mechanisms may be associated with the downregulation of p53 expression and CaM activity along with inhibition of myocardial cell apoptosis and myocardial fibrosis.

Persistence of elevated levels of thyroid hormones in the serum for a long time may result in cardiac overload leading to arrhythmia, cardiac hypertrophy, and myocardial fibrosis [7]. TT3 and TT4 levels are specific and sensitive indicators of hyperthyroidism. In the present study, the hyperthyroid rats showed fast eating and drinking, increased frequency of defecation and urination, slow weight gain, irritability, and frequent activities when compared to the rats of the CON group. The TT3 and TT4 levels of the HHD group of rats also increased when compared to those of the CON group (*P* < 0.05). Examination of the myocardial tissues of the three groups of rats using HE and Masson's staining and electron microscopy revealed that the HHD group exhibited obvious myocardial fibrosis when compared to the CON group. This proved that the rat model of HHD was successfully established, and the findings are consistent with those of the previous studies [8].

It has been demonstrated that the antioxidant, anti-inflammatory, and anti-free-radical effects of the aqueous extract of SC exert a protective effect against myocardial ischemia [9, 10]. However, only limited studies have investigated whether SC exerts the same cardiac effects on myocardial injury due to hyperthyroidism. Our experimental results demonstrated that the levels of TT3 and TT4 in the HHD group were reduced when compared to those in the SC group (*P* < 0.05). Histopathological examination revealed that the SC group showed significant improvement in myocardial fibrosis and the myocardial structure was similar to that of the CON group. Therefore, SC significantly improved the myocardial tissue structure of hyperthyroid rats.

NT-proBNP is an important indicator for the diagnosis and prognosis of heart failure, left ventricular systolic dysfunction, and left ventricular hypertrophy [11]. HHD resulting from a long-term elevated level of serum thyroxine aggravates in rat myocardium over time manifesting initially as increased myocardial contractility, followed by a decline in the myocardial functions. The myocardial contractility initially increases, while the stroke volume remains high over a long period. When the contractility is reduced, the stroke volume remains high, leading to myocardial hypertrophy, cardiac hypertrophy, and myocardial fibrosis [12]. In the present study, the level of NT-proBNP in the HHD group was significantly different when compared to that in the CON group (*P* < 0.05). Combined with the electron microscopy results, it can be suggested that the level of NT-proBNP might be used to assess the cardiac changes caused by hyperthyroidism. This result is consistent with the previous findings [13–15]. However, the level of NT-proBNP following treatment with SC was lower than that in the HHD group, but it did not return to normal levels. The SC group exhibited significant differences when compared to the CON group (*P* < 0.05) but showed no significant difference when compared to the HHD group. This may be attributed to the rapid accumulation of NT-proBNP in the blood, leading to its higher blood level and slower clearance.

Previous studies have shown that overexpression of p53, a tumor suppressor gene, induces cardiomyocyte apoptosis leading to myocardial fibrosis [16]. In the present study, the expression of p53 protein increased in the HHD group when compared to that in the CON group, indicating that p53 protein might be involved in apoptosis of myocardial cells in rats with HHD having increased myocardial fibrosis. The CaM signal transduction pathway is closely related to cardiac hypertrophy, arrhythmia, and heart failure. It has been reported that CaM activity increases with myocardial and cardiac hypertrophy, and myocardial fibrosis may be suppressed by inhibiting the CaM activity [17, 18]. In the present study, the CaM activity increased in the HHD group when compared to that in the CON group, suggesting that CaM protein exhibits an important role in HHD. Compared to the HHD group, the SC group showed decreased p53 expression and CaM activity, suggesting that the improvement of myocardial function in HHD rats treated with SC may be associated with the inhibition of p53 expression and CaM activity. Our results also revealed that Vs, Vd, S, and sSR of the HHD group were significantly lower than those of the CON group, whereas Vs, Vd, S, sSR, and dSR of the SC group were significantly higher than those of the HHD group. This is consistent with p53 expression and CaM activity, as well as the results of histopathological examination, confirming the success of the model establishment and improvement of cardiomyopathy by SC.

VVI is a more objective and comprehensive technique to evaluate the myocardial function when compared to conventional ultrasonography. It reduces the dependence on angle and limitations of the section and aids in automatically tracing the trajectory of the endocardium. There are several studies using VVI for the human apical four-chamber view, although animal studies are limited to the short aorta or left ventricular long axis view [19–23]. Owing to the small size of the rat heart, conventional ultrasound scanning is difficult, and not many studies have been performed on the four-chamber view of rats. VVI may be used to evaluate myocardial function in rats with HHD.

SC fruits contain a number of compounds, such as lignans and organic acids. Schisandrin B is the most abundant lignin exerting beneficial effects on myocardial infarction following myocardial remodeling [24, 25]. Although these results combined with the results of our experiment are encouraging, further investigations are required to determine whether the antioxidant and antifibrotic effects of Schisandrin B are beneficial for the treatment of myocardial fibrosis induced by long-term elevated thyroid hormone levels. However, as the SC used in our experiment was a mixture, the antioxidant and antifibrotic effects of the specific components of SC on hyperthyroidism need to be investigated in further studies.

## 5. Conclusions

Our findings showed that SC significantly improved myocardial fibrosis in rats with HHD, and VVI played an important role in the evaluation of the myocardial function in these rats. SC significantly improved myocardial fibrosis in rats with HHD. VVI might be a valuable tool for the evaluation of myocardial function in these rats.

## Figures and Tables

**Table 1:** Velocity vector imaging parameters of the groups of rats (*n* = 10).

Parameters	CON	HHD	SC
Vs (cm/s)	0.270 ± 0.132	0.102 ± 0.050^b^	0.292 ± 0.145^d^
Vd (cm/s)	0.248 ± 0.108	0.119 ± 0.064^a^	0.288 ± 0.129^d^
S (%)	8.851 ± 6.887	1.604 ± 1.178^b^	6.492 ± 3.674^c^
sSR (1/s)	1.222 ± 0.864	0.512 ± 0.290^a^	1.287 ± 0.781^c^
dSR (1/s)	1.270 ± 1.033	0.516 ± 0.328^a^	1.266 ± 0.684^c^

The results are represented as the mean ± standard deviation (SD). ^a^*P* < 0.05 vs. CON group, ^b^*P* < 0.01 vs. CON group, ^c^*P* < 0.05 vs. HHD group, ^d^*P* < 0.01 vs. HHD group (CON, control group; HHD, hyperthyroidism heart disease model group; SC, *Schisandra chinensis* group; Vs, systolic velocity; Vd, diastolic velocity; S, strain; sSR, systolic strain rate; dSR, diastolic strain rate).

## Data Availability

The data used to support the findings of this study are available from the corresponding author upon request.

## References

[1] Reddy V., Taha W., Kundumadam S., Khan M. (2017). Atrial fibrillation and hyperthyroidism: a literature review. *Indian Heart Journal*.

[2] Osuna P. M., Udovcic M., Sharma M. D. (2017). Hyperthyroidism and the heart. *Methodist DeBakey Cardiovascular Journal*.

[3] Badran H. M., Soltan G., Hassan H. (2012). Changes in left atrial deformation in hypertrophic cardiomyopathy: evaluation by vector velocity imaging. *Global Cardiology Science &amp; Practice*.

[4] McCandless R. T., Minich L. L., Wilkinson S. E., McFadden M. L., Tani L. Y., Menon S. C. (2013). Myocardial strain and strain rate in Kawasaki disease. *European Heart Journal—Cardiovascular Imaging*.

[5] Kim E. Y., Baek I.-H., Rhyu M. R. (2011). Cardioprotective effects of aqueous Schizandra chinensis fruit extract on ovariectomized and balloon-induced carotid artery injury rat models: effects on serum lipid profiles and blood pressure. *Journal of Ethnopharmacology*.

[6] Shen Z., Geng Q., Huang H. (2019). Antioxidative and cardioprotective effects of *Schisandra chinensis* bee pollen extract on isoprenaline-induced myocardial infarction in rats. *Molecules*.

[7] Szkudlarek A. C., Aldenucci B., Miyagui N. I. (2014). Short-term thyroid hormone excess affects the heart but does not affect adrenal activity in rats. *Arquivos brasileiros de cardiologia*.

[8] Ragone M. I., Bonazzola P., Colareda G. A., Consolini A. E. (2015). Cardioprotective effect of hyperthyroidism on the stunned rat heart during ischaemia-reperfusion: energetics and role of mitochondria. *Experimental Physiology*.

[9] Thandavarayan R. A., Giridharan V. V., Arumugam S. (2015). Schisandrin B prevents doxorubicin induced cardiac dysfunction by modulation of DNA damage, oxidative stress and inflammation through inhibition of MAPK/p53 signaling. *PLoS One*.

[10] Sun J. H., Liu X., Cong L. X. (2017). Metabolomics study of the therapeutic mechanism of *Schisandra chinensis* lignans in diet-induced hyperlipidemia mice. *Lipids in Health and Disease*.

[11] Hernández-Romero D., Jover E., Martínez C. M. (2015). TWEAK and NT-proBNP levels predict exercise capacity in hypertrophic cardiomyopathy. *European Journal of Clinical Investigation*.

[12] Runte K. E., Bell S. P., Selby D. E. (2017). Relaxation and the role of calcium in isolated contracting myocardium from patients with hypertensive heart disease and heart failure with preserved ejection fraction. *Circulation: Heart Failure*.

[13] Gu L.-Q., Zhao L., Zhu W. (2011). Relationships between serum levels of thyroid hormones and serum concentrations of asymmetric dimethylarginine (ADMA) and N-terminal-pro-B-type natriuretic peptide (NT-proBNP) in patients with Graves’ disease. *Endocrine*.

[14] Arikan S., Tuzcu A., Gokalp D., Bahceci M., Danis R. (2007). Hyperthyroidism may affect serum N-terminal pro-B-type natriuretic peptide levels independently of cardiac dysfunction. *Clinical Endocrinology*.

[15] Muthukumar S., Sadacharan D., Ravikumar K., Mohanapriya G., Hussain Z., Suresh R. V. (2016). A prospective study on cardiovascular dysfunction in patients with hyperthyroidism and its reversal after surgical cure. *World Journal of Surgery*.

[16] Qi X., Han J., Zhao P., Dong X., Gong S. (2016). S100A4 and P53 in myocardial collagen fibers of hypertrophic cardiomyopathy. *Herz*.

[17] Kreusser M. M., Lehmann L. H., Wolf N. (2016). Inducible cardiomyocyte-specific deletion of CaM kinase II protects from pressure overload-induced heart failure. *Basic Research in Cardiology*.

[18] Li C., Liu S., Zhang J., Liu M., Li J. (2012). Effect of chronic administration of L-thyroxine on Ca^2+^/calmodulin-dependent protein kinase II in rat myocardium. *Chinese Journal of Pathophysiology*.

[19] Yang Z.-R., Zhou Q.-C., Lee L. (2012). Quantitative assessment of left ventricular systolic function in patients with coronary heart disease by velocity vector imaging. *Echocardiography*.

[20] Yurdakul S., Erdemir V. A., Tayyareci Y., Yildirimturk O., Salih Gurel M., Aytekin S. (2013). Subclinical left and right ventricular systolic dysfunction in behcet’s disease: a combined tissue Doppler and velocity vector imaging study. *Journal of Clinical Ultrasound*.

[21] Azam S., Desjardins C. L., Schluchter M. (2012). Comparison of velocity vector imaging echocardiography with magnetic resonance imaging in mouse models of cardiomyopathy. *Circulation: Cardiovascular Imaging*.

[22] Zeng S., Jiang T., Zhou Q. C., Yuan L., Zhou J. W., Cao D. M. (2014). Time-course changes in left ventricular myocardial deformation in STZ-induced rabbits on velocity vector imaging. *Cardiovasc Ultrasound*.

[23] Zhang H., Wei Z., Zhu X. (2014). Assessment of left ventricular myocardial systolic acceleration in diabetic rats using velocity vector imaging. *Journal of Ultrasound in Medicine*.

[24] Chen P., Pang S., Yang N. (2013). Beneficial effects of schisandrin B on the cardiac function in mice model of myocardial infarction. *PLoS One*.

[25] Chun J. N., Cho M., So I., Jeon J.-H. (2014). The protective effects of *Schisandra chinensis* fruit extract and its lignans against cardiovascular disease: a review of the molecular mechanisms. *Fitoterapia*.

